# EUS-guided gastroenterostomy for proximal jejunal obstruction: technique modification for more distal upper GI tract obstruction

**DOI:** 10.1016/j.vgie.2022.08.025

**Published:** 2022-12-08

**Authors:** Abid T. Javed, Ali Abbas

**Affiliations:** Division of Digestive Diseases and Nutrition, Department of Internal Medicine, University of South Florida, Tampa, Florida

**Keywords:** EUS-GE, EUS-guided gastroenterostomy, GOO, gastric outlet obstruction, LAMS, lumen-apposing metal stent

## Abstract

Video 1 Demonstration of the EUS guided gastroenterostomy.

Demonstration of the EUS guided gastroenterostomy.

## Introduction

EUS-guided gastroenterostomy (EUS-GE) using lumen-apposing metal stents (LAMSs) has emerged as a safe, minimally invasive modality for the treatment of gastric outlet obstruction (GOO).^1^ Several techniques have been described, including anterograde, retrograde, and balloon-assisted approaches.^1^, ^2^, ^3^ The “direct” anterograde method of EUS-GE using a nasobiliary catheter to instill contrast material within the target jejunal loop distal to the obstruction uses fewer steps than other approaches and does not require use of a balloon.^4^ However, obstructions in the distal duodenum and proximal jejunum pose a challenge as standard wires and endoscopes may not be able to access the target loop of small bowel for contrast to provide a suitable target for LAMS placement. We describe a case whereby a distal obstruction was bypassed with a simple technique modification.

## Case description

**Figure 1:**
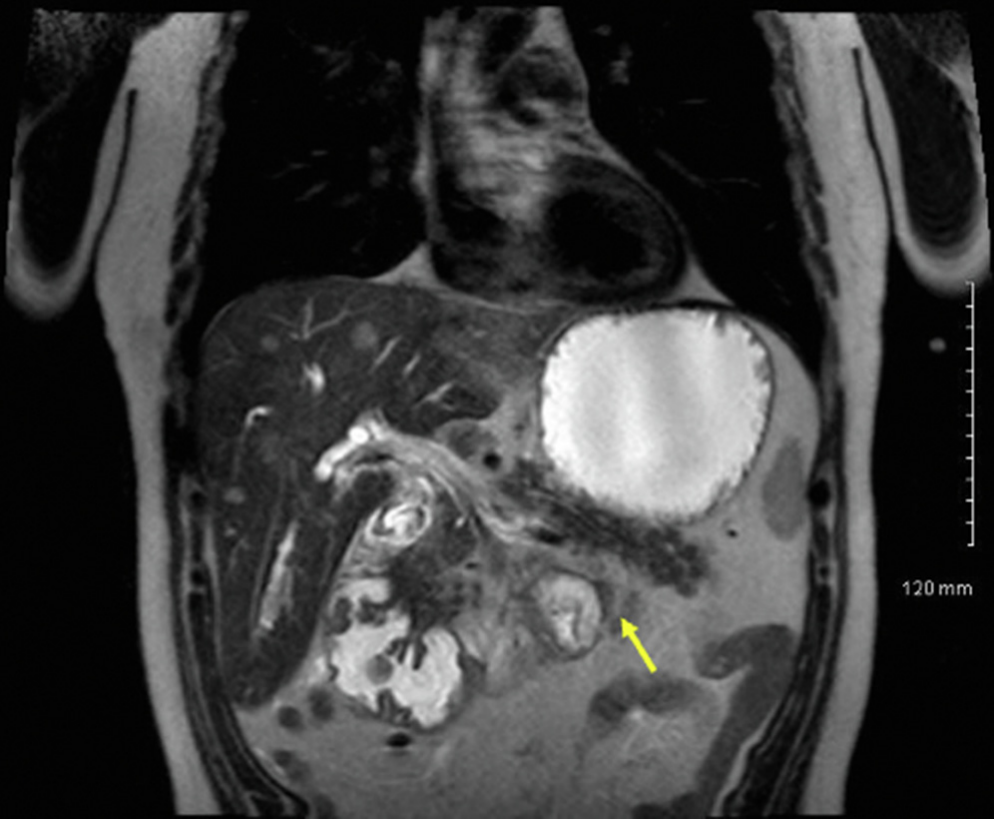
MRI scan of abdomen showing circumferential obstructing mass in the proximal jejunum (*arrow*).

**Figure 2:**
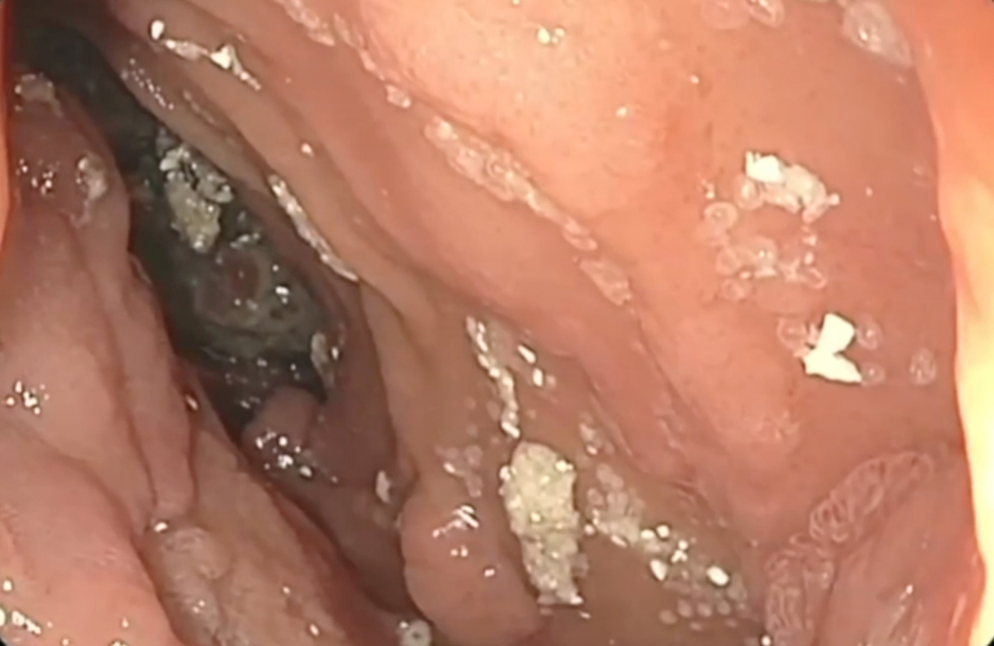
Endoscopic image depicting malignant obstruction in proximal jejunum.

A 62-year-old man with Gardner syndrome presented with symptomatic GOO and weight loss. Push enteroscopy demonstrated an obstructing proximal jejunal polyp (Fig. 1); biopsies demonstrated adenocarcinoma (Fig. 2). The patient was not a surgical candidate because of medical comorbidities and hepatic metastases, so EUS-GE was offered using a modified technique to bypass the relatively distal obstruction.

**Figure 3:**
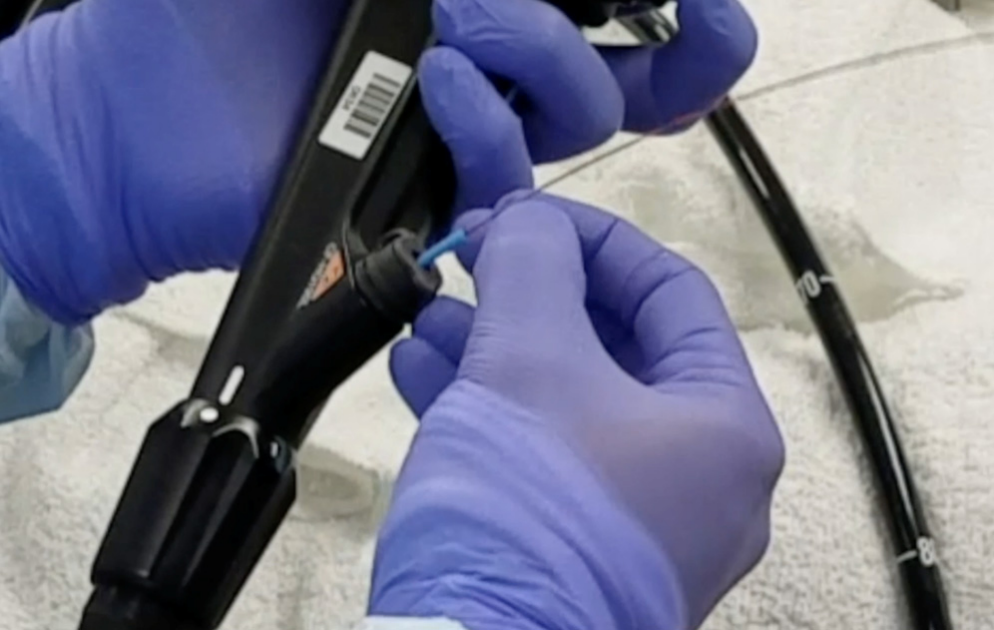
A nasobiliary tube is hubbed over a guidewire that was passed beyond the malignant obstruction.

**Figure 4:**
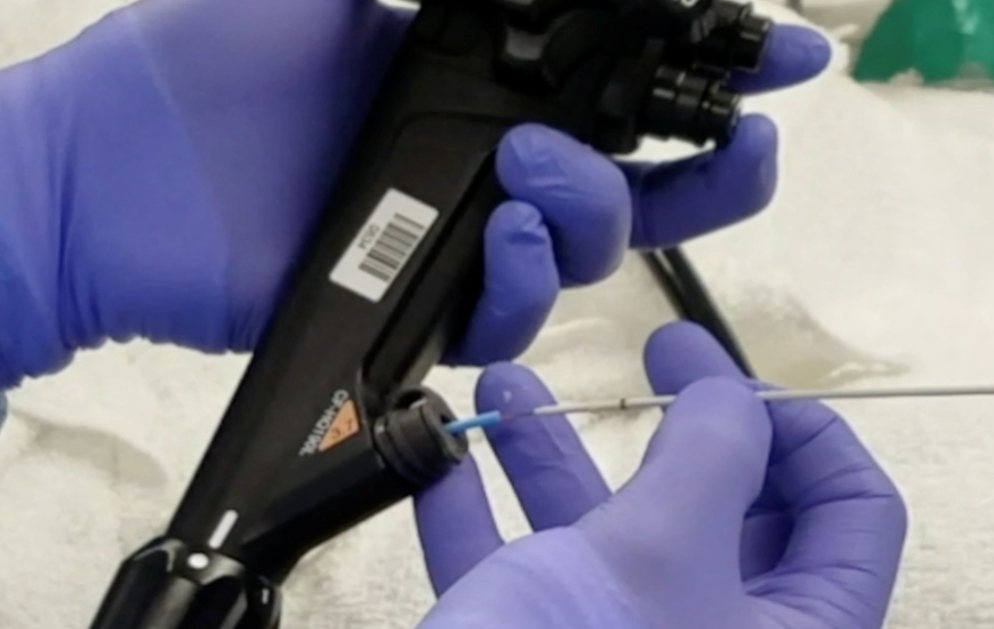
A balloon extraction catheter is passed over the 600-cm wire as a pusher to facilitate removal of the colonoscope while maintaining wire control.

The patient fasted overnight. The procedure was done with the patient under general anesthesia to protect the airway as intraluminal contrast instillation would be used. Five hundred milligrams of intravenous levofloxacin was given intraoperatively prior to LAMS deployment. Using an adult colonoscope, we passed an ERCP cannula over a 600-cm guidewire under fluoroscopic guidance beyond the proximal jejunal obstruction. The intraluminal location of the wire was confirmed via contrast instillation, and then the cannula was withdrawn. An 8.5F nasobiliary catheter was passed over-the-wire in its entirety through the adult colonoscope and was able to reach the jejunal loops distal to the obstruction (Fig. 3). To maintain the position of the nasobiliary catheter during withdrawal of the colonoscope, a 9- to 12-mm ERCP extraction-balloon catheter was advanced over the wire as a pusher with the nasobiliary catheter still in the channel (Fig. 4). The colonoscope was withdrawn, followed by removal of the 600-cm wire. The nasobiliary catheter was then connected to the irrigation pump.

**Figure 5:**
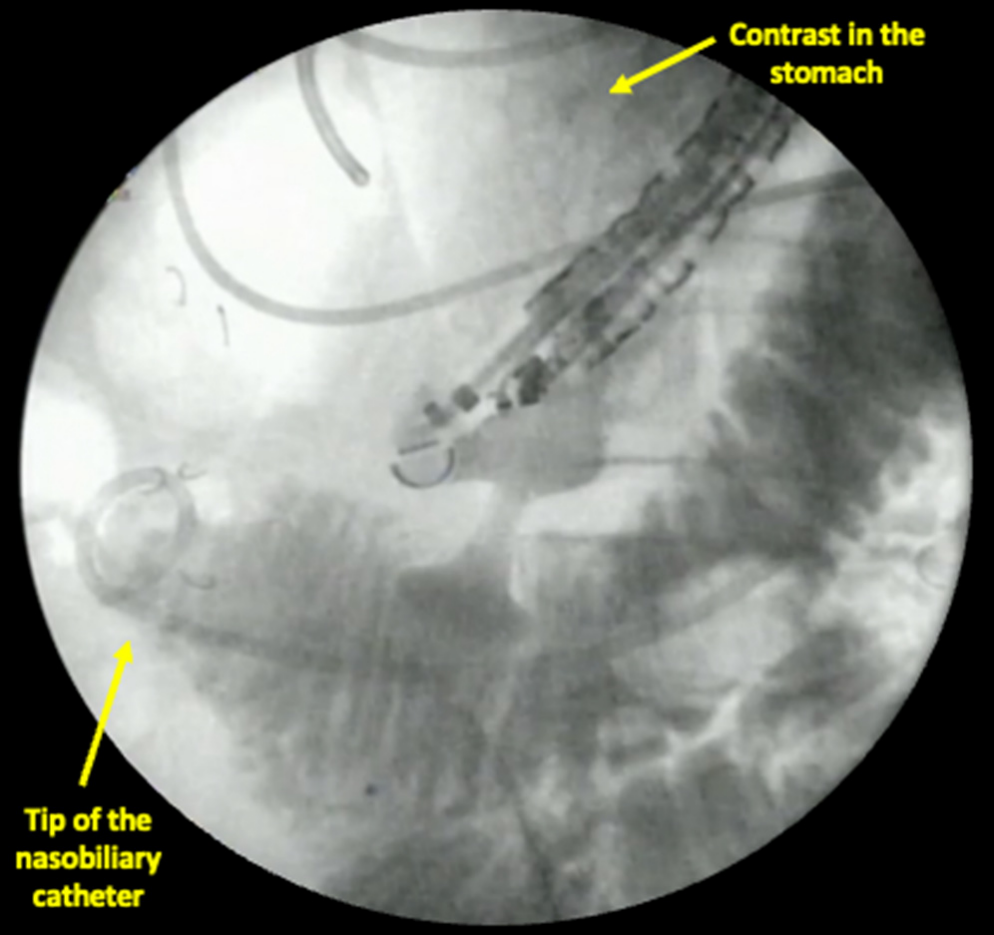
Fluoroscopy shows brisk contrast reflux to the gastric lumen. Carbon dioxide insufflation helps to create a double contrast effect to allow confirmation of a leak and the correct intraluminal placement under fluoroscopy.

Once the nasobiliary catheter is in place, standard direct freehand gastroenterostomy creation can proceed. The linear EUS scope was passed alongside the nasobiliary catheter into the stomach. Five hundred milliliters of diluted contrast was injected intraluminally through the nasobiliary catheter, distal to the obstruction, under sonographic and fluoroscopic guidance to create a suitable target for LAMS deployment. We aimed for a minimum of 20 mm overthrow distance to allow safe deployment of the LAMS. Once a safe target was visualized, a 15-mm LAMS was then deployed in the standard freehanded method. Reflux of contrast into the stomach along with CO2 insufflation (the double-contrast effect) confirmed both correct LAMS placement and absence of a leak (Fig. 5).

**Figure 6:**
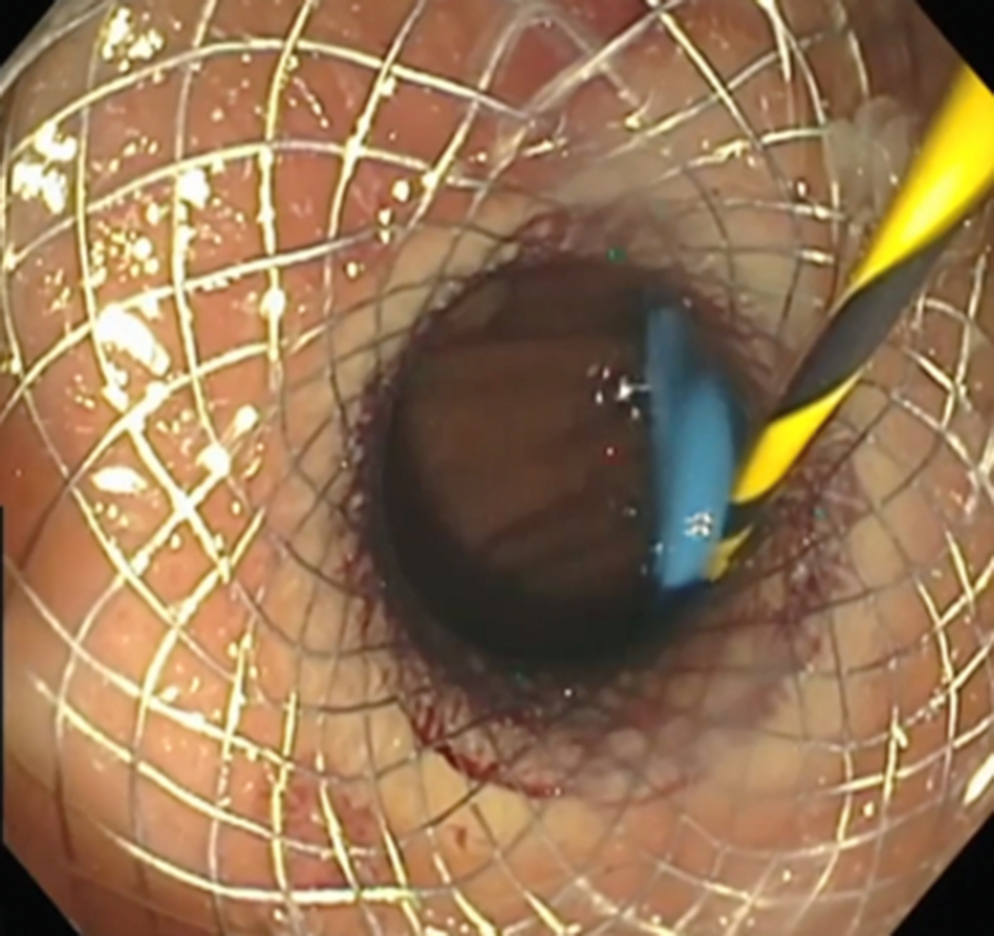
Removal of nasobiliary tube followed by the safety wire.

A 0.035-in × 260-cm guidewire was advanced through the LAMS delivery catheter across the gastroenterostomy into the jejunal lumen distal to the obstruction to maintain access and allow dilation. Then the LAMS catheter was removed over the wire. Finally, over-the-wire through-the-scope balloon dilation to 10 mm of the LAMS was performed. After confirming the correct placement by visualizing the bowel loop endoscopically, the nasobiliary tube was removed followed by the safety wire (Fig. 6). The final location of the gastroenterostomy was in the distal stomach along the greater curvature to facilitate drainage by gravity (Video 1, available online at www.giejournal.org). There were no immediate or delayed procedure-related adverse events.

The patient was placed on a clear liquid diet for the day of the procedure followed by a full liquid diet for 2 weeks postoperatively per our institutional protocol to allow the gastroenterostomy site adequate healing and wall apposition. Subsequently, he was able to advance to a low-residue diet and remained asymptomatic without further admission for the following 12 months. The patient eventually passed away while on hospice. The stent remained in place until the time of death.

## Discussion

The use of a 600-cm wire is critical to access more distal upper GI obstruction and enables the exchange of the colonoscope without running out of wire. Additionally, the use of a longer scope (such as a colonoscope or potentially a small bowel enteroscope) and an ERCP balloon extraction catheter (pusher) were viable techniques to access proximal jejunal obstructions.

## Disclosure

*All authors disclosed no financial relationships.*

## Supplementary data

Video 1 Demonstration of the EUS guided gastroenterostomy.
